# Objective evaluation of major depressive disorder using sleep electroencephalography measured by an in-home portable one-channel device: a preliminary study

**DOI:** 10.1007/s11325-025-03329-9

**Published:** 2025-04-21

**Authors:** Aoi Kawamura, Hiroshi Kadotani, Masahiro Suzuki, Makoto Uchiyama, Naoto Yamada, Kenichi Kuriyama

**Affiliations:** 1 Department of Psychiatry, Shiga University of Medical Science, Otsu, Japan; 2 Department of Sleep-Wake Disorders, National Center of Neurology and Psychiatry, National Institute of Mental Health, Tokyo, Japan; 3 Department of Psychiatry, Nihon University School of Medicine, Tokyo, Japan

**Keywords:** Biomarker, Depression, Fast Fourier transformation, In-home examination, Polysomnography, Sleep EEG

## Abstract

**Purpose:**

Decreased delta and increased alpha wave activity during sleep may be specific pathophysiological features of major depressive disorder; however, their usefulness as biomarkers remains unclear. We examined the use of mean alpha and delta wave power value indices during sleep to identify major depressive disorder using a portable electroencephalography device.

**Methods:**

We compared the mean alpha and delta wave power value indices of six unmedicated patients with major depressive disorder and seven age- and sex-matched healthy controls using a portable electroencephalography device in this case-controlled study.

**Results:**

The ratio of the mean alpha power values for the non-rapid and rapid eye movement periods was significantly lower in the major depressive disorder group (1.3 ± 0.2) than in the healthy group (2.3 ± 0.6; P = 0.004). The ratio of the mean delta power values for the non-rapid eye movement and rapid eye movement periods did not differ between groups but negatively correlated significantly with the Hamilton Rating Scale for Depression score (r = -0.784, P = 0.002). The area under the receiver operating characteristic curve (95% confidence interval) of the mean alpha power ratio for non-rapid eye movement and rapid eye movement periods for distinguishing the two groups was 0.93 (0.78–1.00), and both sensitivity and specificity exceeded 85% at a cut-off value ≤ 1.71.

**Conclusion:**

The alpha- and delta-related power value indices may capture different aspects of major depressive disorder pathology.

## Introduction

Major depressive disorder (MDD) is highly prevalent and causes significant social loss. A longer duration of untreated MDD may predict more frequent relapses, higher chronicity risk, lower remission rates, and worse response to antidepressant treatment [1]. The lack of clinical MDD biomarkers hampers early detection of depressive episodes; therefore, the development of simple and useful MDD biomarkers is required.

More than 80% of patients with MDD have subjective sleep complaints, mostly related to insomnia, with 15–35% having hypersomnolence [2]. Sleep deterioration in MDD may be a risk factor, prodromal or early symptom, and residual MDD symptom [3]. Hence, establishing sleep-related biomarkers for MDD may facilitate early detection.

Since the 1980 s, studies have attempted to extract features reflecting MDD pathophysiology from sleep electroencephalographs (EEG) to utilize them diagnostically, but without any success due to sensitivity/specificity and simplicity of biomarker use.

First, polysomnography (PSG) studies have linked shortened rapid-eye movement (REM) latency, increased REM density, and shortened slow-wave sleep to MDD [4], though these features can also result from conditions like insufficient sleep and excessive time in bed, narcolepsy, anxiety disorders, and schizophrenia. Quantitative EEG (qEEG) with fast Fourier transformation (FFT), which more quantitatively represents sleep microarchitecture than visual analysis of sleep stages [5], revealed decreased delta power in patients with MDD [6], even in remitted cases[7], and may predict depressive episode recurrence more robustly than REM latency [8]. Alpha-wave intrusion in delta-wave sleep (alpha–delta sleep) occurs in MDD significantly correlates with daytime sleepiness [9], suggesting distinctive alpha and delta wave distributions in non-REM (NREM) sleep in patients with MDD. These findings may explain the distribution of spectral compositions of REM and NREM sleep in MDD pathophysiology.

Second, the limited availability of PSG facilities with cumbersome procedures may limit clinical research data collection. Most studies involve medicated or stable MDD patients, as overnight PSG in a medical setting can be burdensome. Additionally, unfamiliar sleep environments and the conditions of PSG examination may affect sleep EEG results[10, 11].

Portable EEG devices can overcome these issues by allowing non-invasive and simple sleep EEG recording. We investigated the use of sleep EEG features, including delta and alpha power values during REM and NREM sleep, derived from an in-home, portable, one-channel EEG device, to distinguish between untreated, unmedicated patients with MDD and healthy individuals.

## Methods

### Participants

We used secondary data from a clinical study (NCT03133013) with 24 participants enrolled between May 2017 and May 2019, all of whom consented to potential future use of their data in mental disorder studies. Using an opt-out procedure for informed consent, six patients with MDD and seven age- and sex-matched healthy participants were enrolled. Case report forms and EEG data were onerously transferred to SleepWell Co., Ltd. (Osaka, Japan) for a project supported by the Japan Agency for Medical Research and Development in which this study was conducted as part.

All patients were untreated, unmedicated, and diagnosed as having MDD by a psychiatric specialist based on a clinical interview using Diagnostic and Statistical Manual of Mental Disorders, Fifth Edition criteria and score ≥ 15 on the clinician-administered Hamilton Rating Scale for Depression (HAM-D). In addition to organic brain disorders, the clinical interview also excluded medical conditions resulting in depressive symptoms or sleep disturbances. However, anxiety disorders are often comorbid and may share a common pathophysiology with MDD, thus allowing them to be accepted as comorbid conditions. Participants who were taking medication that could affect sleep EEG were also excluded. Healthy individuals were unmedicated and free of psychiatric disorders based on a similar assessment.

All participants underwent three-night EEG measurements using a portable, one-channel device at their residence. Data from the second night were prioritized for analysis, followed by the third and first nights, as the first night served as an adaptation, and data dropouts were expected to be lower on the second night than on the third night.

The present study was conducted in accordance with the Declaration of Helsinki. All procedures were approved by the Ethics Committee of Shiga University of Medical Science (#29–195).

### Portable one-channel EEG

Sleep EEGs were obtained using SleepScope (SS), a portable 1-channel EEG device from SleepWell Co., Ltd [12]. Electrodes were placed on the forehead and left mastoid for bipolar derivation, with data recorded in European Data Format at a sampling rate of 128 Hz and filter settings of 0.5–64 Hz, meeting JIS T1203 standards. Data were manually uploaded to the SEAS-G cloud service, which performed EEG spectral analysis through an automated system. This system's sleep staging accuracy aligns with PSG measurements, with kappa values of 0.62–0.67, ensuring reliable sleep EEG sampling.[13].

### Delta and alpha band-related power values

Sleep EEG was deconstructed into frequency components by FFT. Mean power values for delta (0.5–2 Hz) and alpha (8–12 Hz) bands were calculated for 30-s epochs during NREM and REM sleep, along with their NREM/REM ratios. The calculated power value indices were NREM delta mean power (NDMP), REM delta mean power (RDMP), delta mean power ratio (DMPR), NREM alpha mean power (NAMP), REM alpha mean power (RAMP), and alpha mean power ratio (AMPR). The mean delta- or alpha-band-related power values of the total sleep period (NREM + REM) were calculated and defined as TOTAL DELTA and TOTAL ALPHA, respectively. The indices were compared to determine whether they were useful for distinguishing between patients and healthy individuals.

### Statistical analyses

Each statistical method was applied after testing for normality using the Shapiro–Wilk test. Student’s *t*-tests or Mann–Whitney *U*-tests were used to examine differences in continuous data and Fisher’s exact tests to examine differences in qualitative data between groups. For multiple comparisons, Bonferroni correction was applied. Spearman’s rank correlation coefficients were calculated between the power value indices and presence of MDD. Coefficients of correlation between the power value indices and HAM-D total score were similarly calculated. The specificity and sensitivity of the power value indices to identify patients with MDD were analyzed from receiver operating characteristic (ROC) curves. The optimal power value index cut-off values were determined using Youden’s index [14].

All statistical analyses were performed using SPSS v.25 (IBM, Armonk, NY). P-values < 0.05 were considered significant. For multiple comparisons, statistical significance was achieved only at the P < 0.00625 level (Bonferroni corrected: 0.05/8 = 0.00625).

## Results

The two groups had similar age, sex, representative sleep indices, and sleep stage length (Table 1).

**Table 1:** Background information of participants

	Patient group (N = 6)	Healthy group (N = 7)
Age (years), mean (SD)	41.17 (7.08) ^n.s^	41.43 (7.91) ^n.s^
Male, N (%)	2 (33.33%) ^n.s^	1 (14.29%) ^n.s^
TST (min), mean (SD)	408.25 (75.90) ^n.s^	358.57 (50.00) ^n.s^
SL (min), mean (SD)	27.00 (21.31) ^n.s^	14.36 (9.50) ^n.s^
RSL (min), mean (SD)	58.17 (33.31) ^n.s^	70.21 (12.96) ^n.s^
WASO (min), mean (SD)	45.83 (31.02) ^n.s^	43.00 (26.08) ^n.s^
stage N1 (min), mean (SD)	26.50 (9.00) ^n.s^	26.86 (10.90) ^n.s^
stage N2 (min), mean (SD)	262.50 (59.14) ^n.s^	232.43 (47.97) ^n.s^
stage N3 (min), mean (SD)	6.92 (14.14) ^n.s^	4.71 (9.74) ^n.s^
stage R (min), mean (SD)	112.33 (28.18) ^n.s^	94.57 (15.37) ^n.s^

n.s.: The distribution differences were not significantly different between the two groups based on the *t*-test or Fisher’s exact test; the age difference between the two groups was tested using the Mann–Whitney U-test. N, number; SD, standard deviation; TST, total sleep time; SL, sleep latency; RSL, rapid eye movement sleep latency; WASO, wake after sleep onset

**Fig. 1:**
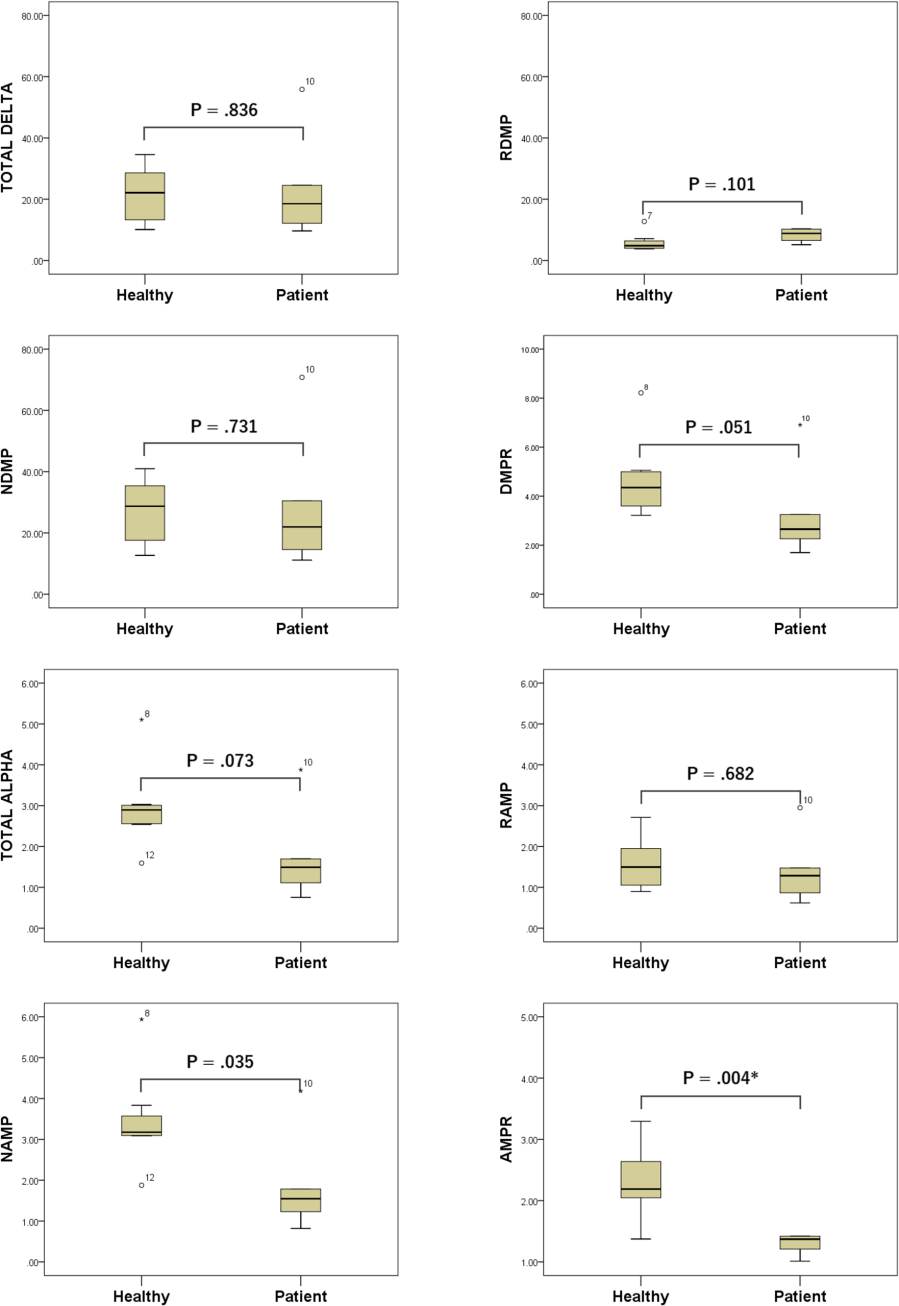
Differences between the healthy and patient groups in alpha-band-related and delta-band-related power value indices. Differences between the two groups were tested using the *t*-test or Mann–Whitney *U*-test. *Significant at the P = 0.00625 level (two-tailed) after Bonferroni correction. The numbers near the outliers in the figures indicate the subject numbers

Differences between the groups in delta- and alpha-band-related power value indices are summarized in Fig. 1. Only AMPR maintained a significant difference after Bonferroni correction and was significantly smaller in the patient group (1.3 ± 0.2) than in the healthy group (2.3 ± 0.6, P = 0.004). NAMP was significantly lower in the patient group (median = 1.55) than in the healthy group (median = 3.18) only before Bonferroni correction (P = 0.035).

AMPR was significantly negatively correlated with MDD diagnosis (r = − 0.742, P = 0.004) even after Bonferroni correction. NAMP and DMPR were significantly correlated with MDD diagnosis (r = − 0.619, P = 0.024; r = − 0.577, P = 0.039) only before Bonferroni correction. A similar trend was observed between the HAM-D total score and power value indices (Table 2). However, significant negative correlations of NAMP and DMPR with HAM-D total scores remained even after Bonferroni correction (r = − 0.784, P = 0.002; r = − 0.784, P = 0.002). TOTAL ALPHA showed trends for a negative correlation with the HAM-D total score (r = − 0.671, P = 0.012) only before Bonferroni correction. AMPR showed the highest correlation coefficient among the power value indices, both for MDD diagnosis and the HAM-D total score (Table 2).

**Table 2:** Spearman rank correlation with Bonferroni correction between power value indices and the diagnostic status or the depressive severity (N = 13)

Power value indices	Healthy/patient (DSM- 5)		HAM-D score	
	r_s_	P	r_s_	P
TOTAL DELTA	-.082	.789	-.197	.519
RDMP	.495	.086	.506	.077
NDMP	-.124	.687	-.237	.435
DMPR	-.577	.039	-.784	.002*
TOTAL ALPHA	-.536	.059	-.671	.012
RAMP	-.206	.499	-.304	.313
NAMP	-.619	.024	-.784	.002*
AMPR	-.742	.004*	-.828	.000*

Patients were scored as s and healthy individuals were scored as 0. *With Bonferroni corrections, correlation was significant at the 0.00625 level (two-tailed). N, number; DSM- 5, The Diagnostic and Statistical Manual of Mental Disorders Fifth Edition; HAM-D, Hamilton Rating Scale for Depression; RDMP, REM delta mean power; NDMP, NREM delta mean power; DMPR, delta mean power ratio; RAMP, REM alpha mean power; NAMP, NREM alpha mean power; AMPR, alpha mean power ratio

**Fig. 2:**
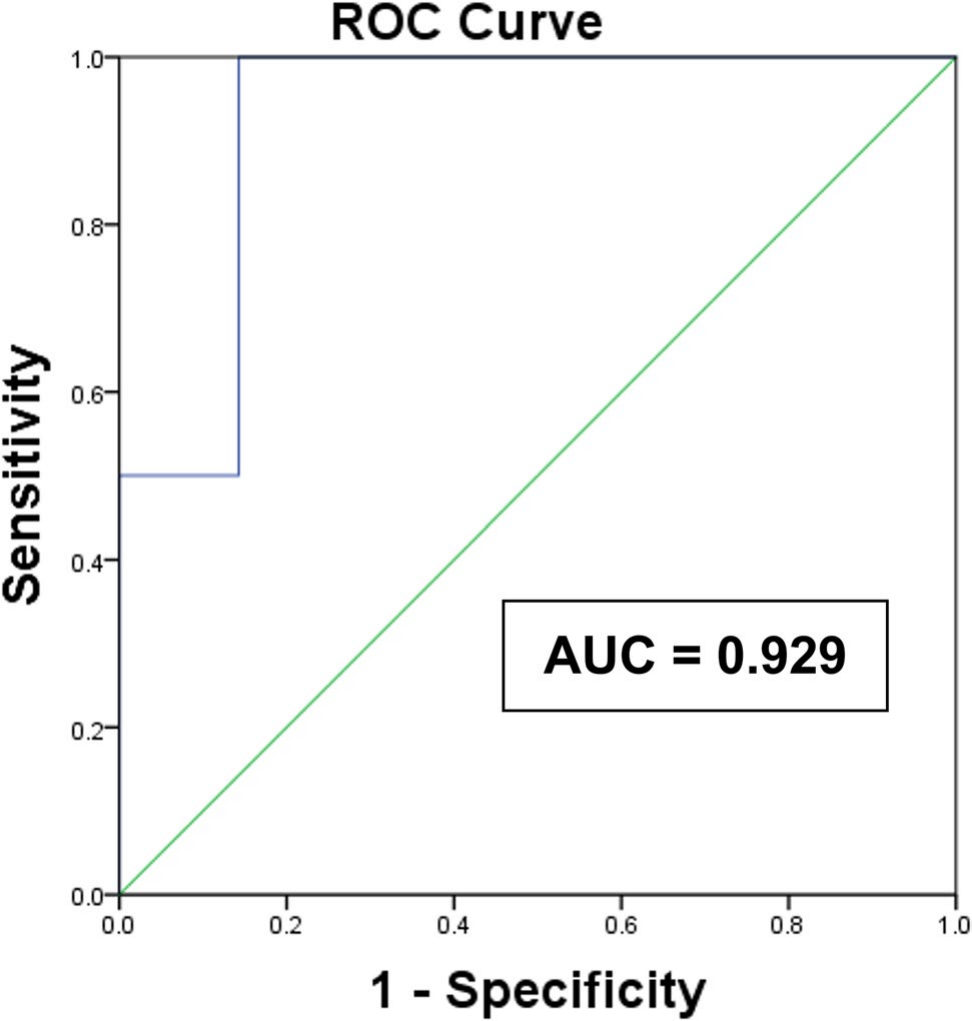
ROC curve analysis for the ratio of the mean alpha power values for non-rapid and rapid eye movement periods (AMPR) for differentiating individuals with major depressive disorder and healthy individuals. ROC, receiver operating characteristic

The mean alpha band-related power value indices appeared to be lower overall in males but was not statistically significant (all P > 0.39). The area under the ROC curve (95% confidence interval) to discriminate the groups was 0.929 (0.777–1.000) (Fig. 2). At the AMPR cut-off ≤ 1.71, determined based on the Youden index, the sensitivity and specificity for MDD detection were 100% and 85.7%, respectively.

## Discussion

AMPR can be a candidate EEG biomarker for depression and reflects MDD features with high sensitivity and specificity. Moreover, it correlated significantly with MDD diagnosis and severity, whereas DMPR correlated significantly only with MDD severity. Alpha- and delta-related power value indices may thus capture different aspects of MDD pathophysiology and could be potential trait and state markers, respectively.

Results suggest that the ratio of NREM-related to REM-related indices is more important than REM- or NREM-related indices alone for differentiating MDD. Sex differences in mean alpha power values during the REM sleep period were not significant owing to small sample size and variability; however, mean alpha power values were generally lower in males, consistent with previous studies [15]. Hence, AMPR may offset sex and individual differences at baseline, focusing on MDD pathology.

As qEEG analysis previously showed reduced all-night delta power in patients with MDD [6], TOTAL DELTA could be lower in the patient group than in the healthy group, but this was not true for this study. Furthermore, although the previous study suggested that alpha-delta sleep is more common in patients with MDD [9], NAMP tended to be rather lower in the MDD patient group in this study. Most previous studies were based on PSG measurements, with different definitions of EEG indices and different EEG measurement sites, hampering comparisons.

AMPR was significantly lower and NAMP tended to be lower in patients with MDD than in healthy participants. Detailed mechanisms, including whether decreases in AMPR in the patient group represent increases or decreases in some brain activity during the NREM sleep period, are currently unclear. The general limitation was the small sample size, which may obscure the characteristics of sleep indices specific to MDD, including REM-related sleep structures in the study.

Alpha- and delta-related power value indices may capture different aspects of MDD pathology and could be potential trait and state markers, respectively, if studied in larger sample sizes.

## Data Availability

Data supporting the results of this study are available upon request from the principal investigator of the study with identification number NCT03133013, which is available on Clinical.Trial.gov.
